# Study on an Air Quality Evaluation Model for Beijing City Under Haze-Fog Pollution Based on New Ambient Air Quality Standards

**DOI:** 10.3390/ijerph110908909

**Published:** 2014-08-28

**Authors:** Li Li, Dong-Jun Liu

**Affiliations:** Shenzhen Graduate School, Harbin Institute of Technology, Shenzhen 518055, China; E-Mail: ximlli@126.com

**Keywords:** air quality, comprehensive evaluation, Ambient Air Quality Standards, entropy weighting method, nearest neighbor method

## Abstract

Since 2012, China has been facing haze-fog weather conditions, and haze-fog pollution and PM_2.5_ have become hot topics. It is very necessary to evaluate and analyze the ecological status of the air environment of China, which is of great significance for environmental protection measures. In this study the current situation of haze-fog pollution in China was analyzed first, and the new Ambient Air Quality Standards were introduced. For the issue of air quality evaluation, a comprehensive evaluation model based on an entropy weighting method and nearest neighbor method was developed. The entropy weighting method was used to determine the weights of indicators, and the nearest neighbor method was utilized to evaluate the air quality levels. Then the comprehensive evaluation model was applied into the practical evaluation problems of air quality in Beijing to analyze the haze-fog pollution. Two simulation experiments were implemented in this study. One experiment included the indicator of PM_2.5_ and was carried out based on the new Ambient Air Quality Standards (GB 3095-2012); the other experiment excluded PM_2.5_ and was carried out based on the old Ambient Air Quality Standards (GB 3095-1996). Their results were compared, and the simulation results showed that PM_2.5_ was an important indicator for air quality and the evaluation results of the new Air Quality Standards were more scientific than the old ones. The haze-fog pollution situation in Beijing City was also analyzed based on these results, and the corresponding management measures were suggested.

## 1. Introduction

With the development of modern industry, industrial equipment and transportation vehicles consume lots of resources and discharge more and more pollutants, and as a result, the atmospheric environment is polluted seriously. Thus the progress of human society is at the expense of the environment [1]. In recent years, areas with polluted air frequently suffer from haze-fog weather conditions in autumn and winter. Especially, in the winter of 2012, a large-scale emergence of haze-fog weather affected the mid-east areas of China, and PM_2.5_, which is one of the key pollution factors in the air environment, became a hot topic in China [2]. As a result the atmospheric environment in China was very harsh, and human health and transportation were seriously affected. The data of the Ministry of Environmental Protection showed that there was large-scale haze-fog pollution in Northeast China, Northwest China, North China, Eastern China and Central China [3,4]. The wide range of haze-fog weather triggered a series of chain reactions, including transportation restrictions, flight delays and increased numbers of patients, *etc*., and people’s life was severely affected [5,6]. In order to protect and improve the living environment and quantitatively analyze the atmospheric environment pollution, new ambient air quality standards were formulated by the Ministry of Environmental Protection [7]. As a result, the “Air Quality Index” (AQI) was introduced to replace the old “Air Pollution Index” (API), and the “Technical Regulations on Ambient Air Quality Index (Trial) (HJ 633-2012)” [8] were published to calculate the AQI.

The severe haze-fog pollution situation has attracted serious concern of academic researchers. Numerous studies have been conducted to investigate the composition, sources, and chemical reactions of the haze-fog pollution in China [9,10,11]. Some scholars claimed the haze-fog pollution was usually accompanied with aerosol concentrations in the atmospheric environment, and had significant impacts on the air quality, human health and visibility [12,13,14]. The formation of haze-fog was very closely linked to atmospheric and meteorological conditions [15,16]. Zhao *et al*. argued that the main source of haze-fog in winter was anthropogenic emissions on a regional scale [17]. Che *et al*. investigated the optical characteristics of the aerosols of haze-fog [18], and Zhang *et al*. analyzed the main chemical components of the aerosols [19]. Wang *et al*. simulated the severe winter of 2013 regional hazes in East Asia and northern China based on simulation models [20].

In order to deal with the haze-fog pollution, the degree of haze-fog pollution should be known first. In this study the ambient air quality was evaluated to investigate the status of haze-fog. Evaluation of ambient air quality is an important part of any atmospheric ecology studies. The assessment of environmental air quality is the process of quantitative description of the ambient air quality by mathematical methods and models. It can help know the present situation and future tendencies of the ambient air quality [21]. Thus, it can provide a scientific basis for the planning and management of ambient air quality. Studies on the evaluation of air quality are an important issue, and have attracted the interest of many scholars. The fuzzy comprehensive evaluation theory was applied in air quality assessment according to the national air quality standard [22]. The level of environmental quality was determined based on the ambient air monitoring data of Dongzhi Country [23]. Artificial neural networks and decision tree models were applied to evaluate the common Air Quality Index in Thessaloniki, Greece [24]. The forest air quality in Yichun Town was evaluated based on BP neural networks [25].

In this study the current situation of haze-fog pollution in China was introduced, and the new ambient air quality standards were analyzed. A comprehensive evaluation model was developed based on an entropy weighting method and nearest neighbor method, and it was applied into the practical problems of air quality in Beijing to analyze the haze-fog pollution status. The entropy weighting method was used to determine the weights of indicators, and the nearest neighbor method was used to evaluate the levels of air quality. Two simulation experiments were implemented. One experiment was with PM_2.5_ and carried out based on the new Ambient Air Quality Standards (GB 3095-2012); the other experiment was without PM_2.5_ and carried out based on the old Ambient Air Quality Standards (GB 3095-1996). We compared their results to investigate the importance of PM_2.5_ and the effects of standards. The situation of haze-fog pollution in Beijing City was then analyzed based on these results.

## 2. Materials

### 2.1. Haze-Fog Pollution in China

Haze-fog events have been hot issues in China since 2012. From the year of 2013 onward haze-fog weather has become more and more serious, and this has caused serious harm to human health, traffic safety, and other production and living aspects of human beings. The main indicator affecting the haze-fog was PM_2.5_, and it became a hot topic that concerned people. The average number of haze-fog days in 2013 was 4.7, which was the largest number recorded in the last 52 years. According to the statistics of the China Meteorological Administration, in mid-east China where the haze-fog weather was the most serious, the average of haze-fog days in the year of 2013 was 35.9. This was equivalent to the fact that there was over one month when the mid-east areas of China were in the shadow of haze-fog, and the ratio of haze-fog days in one year was about 10%.

**Figure 1:**
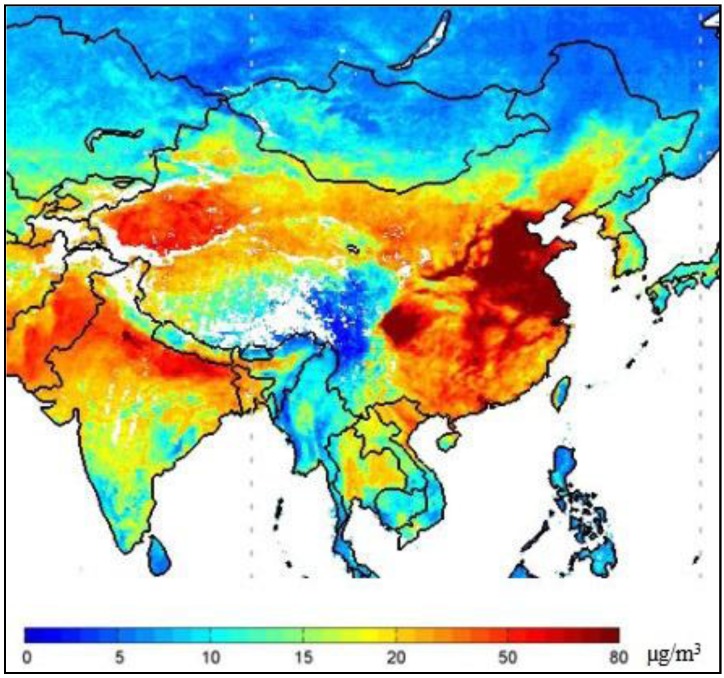
Model map of areas under air pollution based on monitoring data of PM_2.5_.

The PM_2.5_ data in China were monitored through instruments installed on satellites by the National Aeronautics and Space Administration, and the model map of areas under air pollution based on PM_2.5_ monitoring data is shown in Figure 1, where the different colors represent different degrees of PM_2.5_ pollution. The deeper the color of the areas in the map, the heavier the pollution there was. There was continuous haze-fog weather in most parts of China, including Tibet and Xinjiang. The areas with serious haze-fog pollution included the Beijing and Tianjin areas, South Hebei Province, Northeast Henan Province, Western Shandong Province, Jiangsu Province, Anhui Province, Western Zhejiang Province, Northwest Fujian Province, Central Hunan Province, South Jiangxi Province, Central Hubei Province and the Northern Sichuan Basin Area. Southwest China became the only unpolluted land.

The reasons for haze-fog pollution formation are many, and the main reasons can be summarized as follows [26,27]:

(i) The automobile exhaust is the main source of pollutants. In recent years, there are more and more cars in the cities in China and the components in automobile exhaust are the main components of the haze-fog;
(ii) Secondary pollution from factories is also an important reason. There is much benzene and aldehydes in chemical pollution emissions, and they are important components of haze-fog;
(iii) The relative humidity near the ground in the haze-fog areas is relatively high, and the ground has lots of dust, so particulate matter can easily form;
(iv) Burning garbage and burning coal in winter for heating can also generate pollutants.

### 2.2. New Air Quality Standards

In order to protect and improve the living environment and analyze the atmospheric environment pollution quantitatively, new ambient air quality standards were formulated. The standards formulated the function division of air quality, grading standards, pollutant indicators, time to acquire pollutants, concentration limit values, sampling and analysis methods, and effectiveness of data statistics. The air quality standard in effect before 2012 in China was “Ambient Air Quality Standards (GB 3095-1996)” [28]. They were published and implemented in 1996 and modified in 2000. However, because the haze-fog pollution in China is more and more serious, the regulation of the measurement of PM_10_ and PM_2.5_ in ambient air, was published by the Ministry of Environmental Protection, and started to be implemented from 1 January 2011. The measurement of PM_2.5_ was standardized for the first time in the regulation, but it was not included in the mandatory monitoring indicators at that time. In February 2012, the State Council of China issued the new air quality standards: “Ambient Air Quality Standards (GB 3095-2012)” [29]. This regulation will be implemented in 1 January 2016. In this new standard the PM_2.5_ values were mandatorily included. Three methods of PM_2.5_ monitoring were published in the regulation of “PM_2.5_ automatic monitoring instrument specifications and requirements (trial)” published in May 2012.

The threshold values of some main indicators in the Ambient Air Quality Standards (GB 3095-1996) and (GB 3095-2012) are shown in Table 1 and Table 2. The main differences between the new Ambient Air Quality Standards (GB 3095-2012) and the old ones (GB 3095-1996) were as follows:

(i) The three levels of air environment function classification were adjusted to two levels, and the threshold values were adjusted accordingly;
(ii) PM_2.5_ was included in the standards, and 8-hour average threshold values for O_3_ were also included;
(iii) The threshold values of PM_10_ (annual mean) and NO_2_ (1-hour average) were adjusted, and they were more severe than before;
(iv) The regulations of the validity of data statistics were adjusted.

**Table 1:** Threshold values of ambient air pollutant indicators in GB 3095-1996.

No.	Pollutant Indicator	Average Time	Threshold Values			Unit
			Level I	Level II	Level III	
1	SO_2_	Annual mean	20	60	100	µg/m^3^
		24-hour average	50	150	250	
		1-hour average	150	500	700	
2	NO_2_	Annual mean	40	40	80	µg/m^3^
		24-hour average	80	80	120	
		1-hour average	120	120	240	
3	CO	24-hour average	4	4	6	mg/m^3^
		1-hour average	10	10	20	
4	O_3_	1-hour average	120	160	200	µg/m^3^
5	PM_10_	Annual mean	40	100	150	µg/m^3^
		24-hour average	50	150	250	

**Table 2:** Threshold values of ambient air pollutant indicators in GB 3095-2012.

No.	Pollutant Indicator	Average Time	Threshold Values		Unit
			Level I	Level II	
1	SO_2_	Annual mean	20	60	
		24-hour average	50	150	µg/m^3^
		1-hour average	150	500	
2	NO_2_	Annual mean	40	40	
		24-hour average	80	80	µg/m^3^
		1-hour average	200	200	
3	CO	24-hour average	4	4	mg/m^3^
		1-hour average	10	10	
4	O_3_	8-hour average	100	160	µg/m^3^
		1-hour average	160	200	
5	PM_10_	Annual mean	40	70	µg/m^3^
		24-hour average	50	150	
6	PM_2.5_	Annual mean	15	35	µg/m^3^
		24-hour average	35	75	

## 3. Methods

### 3.1. Entropy Weighting Method

In the evaluation system, determination of weights for all the indicators is an important process that can measure the degree of impact of indicators. When the weight of an indicator is high, it has great impact on the capability; otherwise, the impact is lower. In information theory, information entropy is an important concept, and it can measure the amount of useful information that the data provides in a system [30]. The basic criteria of entropy weighting method are as follows: when the data of the multiple evaluated objects on one indicator show great differences, the entropy value of this indicator must be low according to information theory. This shows that this indicator can contribute much useful information and thus the weight of this indicator should be set high; otherwise, when the entropy value of an indicator was high, it may contribute little useful information according to the information theory. Its weight should be correspondingly low [31]. The procedures for weighting the indicators are as follows:

(i) The original data of all indicators should be normalized, and this can eliminate the impact of dimension. For one benefit indicator, the higher its value is, the better its effect on the air quality was. The normalization equation is:

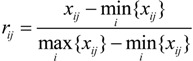
(1)


For a cost indicator, the lower its value is, the better its effect on the air quality is. The normalization equation is:

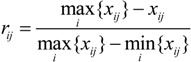
(2)
where, *x_ij_* (*i*=1, 2, …, *m*, and *j* =1, 2, …, *n*) is the monitoring value of the *j*-th object on the *i*-th indicator, and *r_ij_* is the dimensionless value normalized.

(ii) For the evaluation problem with *m* indicators and *n* evaluated objects, the entropy value *p_i_* of the *i*-th indicator can be defined as:

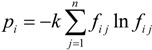
(3)
where 
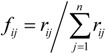
, *k*=1/ln *n*, *i*=1, 2, …, *m*. When *f_ij_*= 0, we set *f_ij_* ln *f_ij_* =0.

(iii) The weight of the *i*-th indicator *λ_i_* can be calculated according to the information entropy theory:

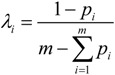
(4)
where 0 ≤ *λ_i_* ≤ 1, and 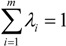.

The entropy weighting method is a good objective weighting method, and it can reflect the degree of effective amount of information provided. In this study the entropy weighting method is used to weight the indicators of the air quality.

### 3.2. Nearest Neighbor Method

The nearest neighbor method is one of the clustering analysis methods. The purpose of the clustering analysis method is to classify the samples with close distances to the clustering centers as the same class based on some criterion. The basic ideas of the nearest neighbor method are as follows: for an evaluation problem with multiple objects, it is supposed that there are multiple samples *x_i_* (*i*=1, 2, …, *N*). For one sample *x_i_* to be classified, we investigate the distances between *x_i_* and x^*^ which is the known clustering center. The cluster of sample *x_i_* is defined as the cluster with the nearest distance from the known cluster [32]. According to this classification ideology, the distance of nearest neighbor method can be set as:

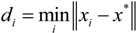
(5)


The Euclidean distance function is employed in this study, and the equation is:

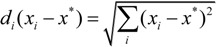
(6)


In the evaluation system, different indicators are often with different dimensions, and the data of indicators should be normalized first. The nearest neighbor method is a practical evaluation method. It is suitable for processing multi-indicator evaluation problems. It has a simple principle, easy calculation and high practicability. When this method was applied to the evaluation work, good comparability assessment results could be obtained.

## 4. Evaluation Model and Algorithm

**Figure 2:**
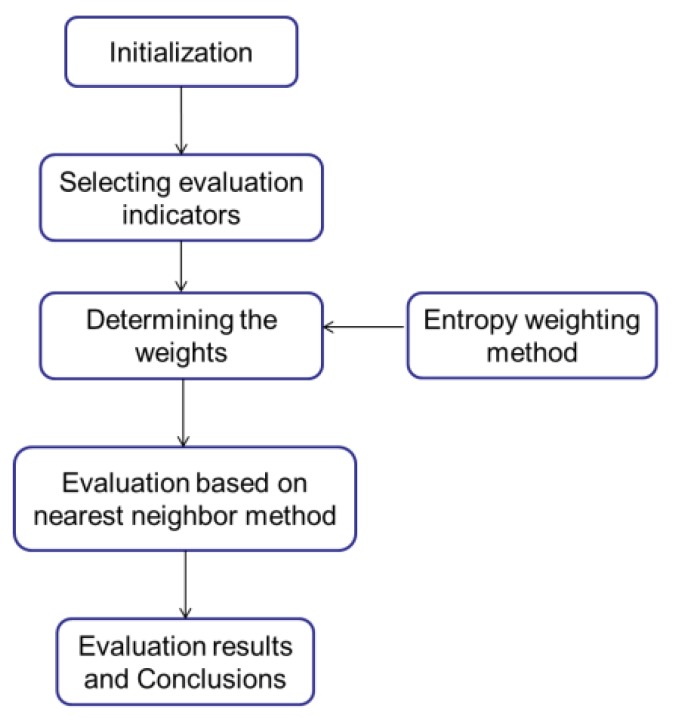
Evaluation model algorithm.

For the issue of air quality evaluation, a comprehensive evaluation model based on the entropy weighting method and nearest neighbor method was developed. The entropy weighting method was used to weight the various air quality indicators, and the importance of each indicator was analyzed. The nearest neighbor method was utilized to evaluate the air quality according to the Ambient Air Quality Standards. The algorithm of the evaluation model is shown in Figure 2.

Then the comprehensive evaluation model was applied to the practical problem of evaluating the air quality in Beijing to analyze the haze-fog pollution status. Because the most important changes of the Ambient Air Quality Standards were the introduction of PM_2.5_, we set two simulation experiments, one is with the indicator of PM_2.5_, and the other not. The simulation experiment with PM_2.5_ was carried out based on the new Ambient Air Quality Standards (GB 3095-2012), and the other experiment without PM_2.5_ was based on the old Ambient Air Quality Standards (GB 3095-1996). We compared their results, and analyzed the air quality of Beijing City at the same time. Thus the ambient air quality of Beijing City was analyzed comprehensively, and it is hoped that the results will provide helpful suggestions for the health and life of the people. The study procedures of this paper were as follows:

Step 1: Initialize, and collect the original data of all indicators of air quality;
Step 2: Define the indicators and their weights. The entropy weighting method was used to determine the weights of all indicators, and their importance was analyzed;
Step 3: Construct the evaluation model based on the nearest neighbor method, and it was used to evaluate the air quality of Beijing City;
Step 4: Use the model to evaluate the air quality in two simulation experiments. One experiment was with PM_2.5_ and carried out based on the new Ambient Air Quality Standards (GB 3095-2012); the other experiment was without PM2.5 and carried out based on the old Ambient Air Quality Standards (GB 3095-1996);
Step 5: Compare the results of the two simulation experiments, and analyze the air quality of Beijing City according to the evaluation results of the model;
Step 6: Draw conclusions, and provide reasonable suggests to decision making according to the research results.

## 5. Results and Analysis

### 5.1. Simulation Experiments

The two simulation experiments are described in this section. One experiment was with PM_2.5_ and carried out based on the new Ambient Air Quality Standards (GB 3095-2012); the other experiment was without PM_2.5_ and carried out based on the old Ambient Air Quality Standards (GB 3095-1996). The original data were the values of some important indicators in February 2014, which was one of the most polluted months in Beijing. The original data, as shown in Table 3, were from the China National Environmental Monitoring Center, and they were 24-hour averages of all pollutants. The indicators which reflected the air quality were PM_2.5_, PM_10_, CO, NO_2_, and SO_2_.

**Table 3:** Air quality data of Beijing city in February 2014.

Date	PM_2.5_ (μg/m^3^)	PM_10_ (μg/m^3^)	CO (mg/m^3^)	NO_2_ (μg/m^3^)	SO_2_ (μg/m^3^)
02–01	135	146	1.99	57	51
02–02	63	93	1.19	30	18
02–03	5	31	0.30	8	4
02–04	26	39	0.62	21	19
02–05	87	101	1.58	48	52
02–06	119	131	2.08	65	67
02–07	94	60	1.45	51	34
02–08	72	43	1.27	38	23
02–09	8	15	0.45	17	15
02–10	23	27	0.74	37	25
02–11	100	114	2.09	78	58
02–12	111	115	2.15	77	53
02–13	190	176	2.85	84	74
02–14	265	295	3.11	94	73
02–15	387	443	3.75	107	102
02–16	296	204	3.18	87	79
02–17	104	50	2.03	71	65
02–18	70	84	1.13	61	37
02–19	66	74	1.23	61	34
02–20	161	171	2.16	78	43
02–21	257	284	2.67	103	77
02–22	262	299	3.32	101	99
02–23	212	248	3.61	92	128
02–24	259	327	4.74	119	133
02–25	353	390	5.29	121	75
02–26	315	250	3.70	105	80
02–27	15	22	0.43	22	12
02–28	77	102	1.50	71	32

Using the formulas in Section 3.1, the weights of all indicators were calculated based on the entropy weighting method according to the data in Table 3 and the results are shown in Table 4.

**Table 4:** Entropy values and weights of indicators.

	PM_2.5_	PM_10_	CO	NO_2_	SO_2_
Entropy value	1.3887	1.3999	1.4002	1.3595	1.3944
Weight	0.2001	0.2059	0.2060	0.1850	0.2030

From Table 4 we could see that the weights of CO and PM_10_ were high, while that of NO_2_ was low. The differences between all indicators were not much, and this indicated that all indicators were very important to the evaluation system.

In next step the nearest neighbor method was used to evaluate the air quality of Beijing City in February. The air quality levels were set as the clustering centers, and we investigated the distances between each sample and the clustering centers. Using the formulas in Section 3.2, we calculated the distances, and the level of air quality was determined according to the principle of minimum distance. Two simulation experiments would be implemented. One experiment was with PM_2.5_ and carried out based on the new Ambient Air Quality Standards (GB 3095-2012); the other experiment was without PM2.5 and carried out based on the old Ambient Air Quality Standards (GB 3095-1996). We compared their results to investigate the importance of PM_2.5_ and the effects of the standards.

In the first simulation experiment based on the new Ambient Air Quality Standards (GB 3095-2012), five indicators were included (PM_2.5_, PM_10_, CO, NO_2_, and SO_2_). The air quality was evaluated and the results, shown in Table 5, were calculated according to the formulas in Section 3.2. In Table 5, *d*_1_ stands for the distances between the daily values of all indicators and the threshold values of Level I, while *d*_2_ stands for the distances from the daily values of all indicators to the threshold values of Level II in the standards (GB 3095-2012). In addition, from Table 2 we could see that the NO_2_ threshold values at Level I and Level II were the same, and so were those of CO, therefore, we could ignore these two indicators, which may reduce the evaluation workload.

**Table 5:** Evaluation results based on Ambient Air Quality Standards (GB 3095-2012).

Date	*d*_1_	*d*_2_	Rank	Date	*d*_1_	*d*_2_	Rank
02–01	0.2949	0.2357	II	02–15	0.7511	0.6146	II
02–02	0.3725	0.3740	I	02–16	0.4535	0.3520	II
02–03	0.4313	0.4695	I	02–17	0.1865	0.2107	I
02–04	0.3616	0.3981	I	02–18	0.2890	0.2945	I
02–05	0.2374	0.2242	II	02–19	0.2993	0.3118	I
02–06	0.2222	0.1624	II	02–20	0.3565	0.2860	II
02–07	0.3072	0.3195	I	02–21	0.4715	0.3435	II
02–08	0.3477	0.3726	I	02–22	0.4801	0.3460	II
02–09	0.3840	0.4328	I	02–23	0.3987	0.2759	II
02–10	0.3361	0.3816	I	02–24	0.5262	0.3954	II
02–11	0.2277	0.1964	II	02–25	0.6732	0.5408	II
02–12	0.2538	0.2209	II	02–26	0.5080	0.3948	II
02–13	0.3108	0.2103	II	02–27	0.3953	0.4385	I
02–14	0.4941	0.3665	II	02–28	0.3173	0.3098	II

In the second simulation experiment, four indicators were included (PM_10_, CO, NO_2_, and SO_2_). PM_2.5_ was excluded. The old Ambient Air Quality Standards (GB 3095-1996) was adopted to evaluate the air quality. The evaluation results are shown in Table 6. In Table 6, *d*_1_ and *d*_2_ also stand for the distances between the daily values of all indicators and the threshold values of Level I and Level II, while *d*_3_ stands for the distances between the daily values of all indicators and the threshold values of Level III in the standards (GB 3095-1996).

**Table 6:** Evaluation results based on Ambient Air Quality Standards (GB 3095-1996).

Date	*d*_1_	*d*_2_	*d*_3_	Rank	Date	*d*_1_	*d*_2_	*d*_3_	Rank
02–01	0.2352	0.2185	0.5498	II	02–15	0.5602	0.3759	0.3075	III
02–02	0.3264	0.4082	0.7463	I	02–16	0.2739	0.0797	0.3365	II
02–03	0.4602	0.5583	0.8978	I	02–17	0.2139	0.2068	0.5293	II
02–04	0.3907	0.4757	0.8148	I	02–18	0.2634	0.3188	0.6491	I
02–05	0.2657	0.2721	0.6063	I	02–19	0.2510	0.3207	0.6531	I
02–06	0.2416	0.1715	0.4977	II	02–20	0.2090	0.2046	0.5154	II
02–07	0.2471	0.3238	0.6625	I	02–21	0.3616	0.2069	0.3545	II
02–08	0.2927	0.3896	0.7306	I	02–22	0.4150	0.2118	0.2602	II
02–09	0.4183	0.5116	0.8511	I	02–23	0.4581	0.2247	0.2093	III
02–10	0.3397	0.4276	0.766	I	02–24	0.5639	0.3584	0.1071	III
02–11	0.2029	0.1771	0.4996	II	02–25	0.5080	0.3789	0.2808	III
02–12	0.1884	0.1828	0.5086	II	02–26	0.3299	0.1601	0.2703	II
02–13	0.2441	0.0825	0.3819	II	02–27	0.4074	0.5056	0.8454	I
02–14	0.3471	0.1894	0.3452	II	02–28	0.2202	0.2893	0.6152	I

### 5.2. Analysis and Discussion

As shown in Table 5, the air qualities of less than half a month in February were at Level I, and those of the other dates were at Level II. The results of Experiment 2 in Table 6 were basically consistent with those of Experiment 1. The days with Level II air quality in Table 5 were at Level II or III in Table 6, while the days with Level I air quality in Table 6 were still at Level I in Table 5.

However, there were some differences between the results of our two simulation experiments. The air qualities on 5 and 28 February were at Level II in Experiment 1 in Table 5, while those were at Level I in Experiment 2 in Table 6. This illustrated the air quality became worse due to the introduction of PM_2.5_. If the air quality were evaluated based on the old Ambient Air Quality Standards (GB 3095-1996), the results tended to be not scientific or deviate from the actual situation. PM_2.5_, as a new indicator that reflects air quality, plays an important role in the evaluation process, and it is receiving more and more attention in China. Thus the new Ambient Air Quality Standards (GB 3095-2012) are more scientific than the old ones.

There were two continuous periods when the air qualities were severe polluted (at Level II in Table 5, or Level II and III in Table 6), that is, from the 11th to the 16th and the 20th to the 26th. The data of all indicators in those days were high, especially the PM_2.5_ indicator, which was one of the most important factors influencing the air quality of Beijing City. The PM_2.5_ concentration on 11 to 16 February in Beijing increased obviously in Table 3, because it was affected by the fireworks during the Spring Festival and adverse weather conditions. On 20 to 26 February, due to the adverse weather conditions, Beijing was experiencing serious haze-fog pollution, and this haze-fog pollution event was wide ranging, of severe degree, and long lasting.

## 6. Conclusions

Haze-fog pollution in China was very severe during autumn and winter in recent years. In order to protect and improve the living environment and analyze the atmospheric environment pollution quantitatively, new ambient air quality standards were issued. A comprehensive evaluation model based on an entropy weighting method and nearest neighbor method was developed according to the new Ambient Air Quality Standards (GB 3095-2012), compared with the old standards (GB 3095-1996). The model was applied into assess the air quality in Beijing in February 2014. The simulation results showed that PM_2.5_ played an important role in the evaluation process, and could affect the air quality to a large extent. The results based on the new Ambient Air Quality Standards (GB 3095-2012) were more scientific than the old standards (GB 3095-1996). In February 2014 in Beijing there were two continuous periods when the air qualities was severe polluted, that is, the 11th to the 16th and the 20th to the 26th. The air quality in Beijing during less than half a month in February was at Level I, and those of the other dates were at Level II according to the new standards. This was affected by the fireworks during the Spring Festival and adverse weather conditions, and the haze-fog pollution situation in Beijing was still severe.

The haze-fog pollution is heavy in China, and the management and control of haze-fog is very important. The prevention of haze-fog pollution is a systematic project. This requires the environmental protection departments to take the lead, and it also needs social consensus to urge the relevant work. Measures from two aspects are suggested to reduce the haze-fog pollution. One is to reduce the pollution emissions from the sources. We must make efforts to adhere to plans to reduce pollutant emissions. The other is to establish emergency plans for heavy pollution weather. The measures can be taken in advance according to the weather forecast in order to reduce or avoid the occurrence of weather-driven heavy pollution.
